# Early biomarkers associated with prenatal alcohol exposure and neurodevelopmental outcomes

**DOI:** 10.3389/fphar.2026.1816529

**Published:** 2026-06-02

**Authors:** Isabel Ochando, Angela Sempere, Daniela Navarro

**Affiliations:** 1 Departamento de Histología y Anatomía, Facultad de Medicina, Universidad Miguel Hernández, Alicante, Spain; 2 Departmento de Neuropediatria, Centre de Teràpia Interfamiliar, HLA Vistahermosa Hospital, Alicante, Spain; 3 Instituto de Neurociencias, Universidad Miguel Hernández-CSIC, Alicante, Spain; 4 Instituto de Investigación Sanitaria y Biomédica de Alicante (ISABIAL), Alicante, Spain

**Keywords:** prenatal alcohol exposure, neurodevelopment, biomarkers, placenta, peripheral biological fluids, epigenetics, neuroinflammation

## Abstract

Prenatal alcohol exposure (PAE) is a major early-life stressor associated with long-lasting neurodevelopmental and neurocognitive alterations. Increasing evidence from human studies indicates that PAE leaves persistent molecular signatures that can be detected in the placenta and in clinically accessible biological fluids. This Mini Review summarizes recent advances in the identification of epigenetic, immune, endocrine, and RNA-based biomarkers measured in placental tissue, umbilical cord blood, and peripheral fluids, and their associations with neurodevelopmental outcomes. We highlight convergent findings across heterogeneous study designs, discuss current methodological challenges and research gaps, and consider future directions toward integrative and longitudinal biomarker approaches. Together, the reviewed evidence supports the potential of placental and circulating biomarkers to improve early risk stratification and to advance understanding of neurodevelopmental alterations following PAE.

## 1 Introduction

Prenatal alcohol exposure (PAE) is widely recognized as one of the leading preventable causes of neurodevelopmental impairment worldwide (Riley et al., 2011). Alcohol readily crosses the placenta and interferes with fundamental processes of brain development, including neurogenesis, neuronal migration, synaptogenesis, and myelination (Guerri et al., 2009; Mattson et al., 2011). The resulting clinical manifestations encompass a broad continuum of cognitive, behavioral, and emotional alterations collectively referred to as fetal alcohol spectrum disorders (FASD) (Jones and Smith, 1973; Mattson et al., 2011).

Epidemiological studies estimate that FASD affects approximately 1%–5% of children in Western countries, although prevalence varies according to diagnostic criteria and population characteristics (Roozen et al., 2016; May et al., 2018). Importantly, neurodevelopmental impairment often occurs in the absence of characteristic facial dysmorphology, contributing to underdiagnosis (Mattson et al., 2011; Cook et al., 2016). Children with PAE often have deficits in executive functioning, attention, learning, emotional regulation, and adaptive behavior. These deficits may become more apparent during the school-age or adolescent years (Mattson et al., 2011; Kingdon et al., 2016).

Large cohort studies have demonstrated associations between PAE and adverse psychological, behavioral, and cognitive outcomes across development (Lees et al., 2020). Moreover, subtle but significant neurobehavioral alterations have been documented during the neonatal period following low-to-moderate exposure, even in the absence of overt physical anomalies or perinatal complications (Maxwell et al., 2025). These findings underscore the vulnerability of the developing brain and the limitations of relying solely on early clinical phenotyping.

A major obstacle in both clinical practice and research is the accurate identification of PAE. Although maternal self-report is the most commonly used screening method, it is subject to recall bias, stigma, and a tendency to underestimate exposure (Ernhart et al., 1988). Objective biomarkers of alcohol exposure measured in placental tissue or umbilical cord blood, such as phosphatidylethanol, have demonstrated higher detection rates than questionnaire-based methods, even when short-term neonatal outcomes appear unaffected (Maxwell et al., 2019). Together, these observations reveal a critical gap between exposure ascertainment, early biological alterations, and later-emerging neurodevelopmental phenotypes.

In parallel, increasing attention has focused on the biological mechanisms through which early-life stressors, including PAE, exert long-lasting effects on brain development. Experimental and human studies converge on the concept of developmental programming, whereby prenatal insults induce stable molecular alterations that persist beyond the exposure window and influence long-term neurodevelopmental trajectories (Gluckman and Hanson, 2004; Monk et al., 2012). This perspective aligns with the Developmental Origins of Health and Disease framework, which emphasizes how early environmental stressors shape later health and neurobiological outcomes (Gluckman and Hanson, 2004).

Importantly, many of these molecular alterations are detectable in placenta and clinically accessible biological fluids, offering a unique opportunity to identify early biomarkers associated with altered neurodevelopment. This Mini Review focuses on recent human studies investigating placental and circulating biomarkers related to PAE and neurodevelopmental outcomes. By integrating evidence across epigenetic, immune, endocrine, vascular, and RNA-based domains, we aim to summarize emerging concepts, identify current gaps, and outline future directions toward translational biomarker strategies in the context of early-life alcohol exposure.

## 2 Early biomarkers associated with prenatal alcohol exposure

Based on evidence that PAE induces persistent biological alterations and that neurodevelopmental manifestations may emerge progressively over time, we reviewed human studies investigating placental and circulating molecular biomarkers associated with neurodevelopmental outcomes following PAE. Our aim was to identify biologically meaningful signatures detectable in clinically accessible tissues that may reflect early neurodevelopmental vulnerability. The characteristics and principal findings of the included studies are summarized in Table 1.

**TABLE 1:** Characteristics and main findings of human studies investigating placental and circulating biomarkers in prenatal alcohol exposure.

Study, year	Study population	Biological sample	Biomarker type	Specific biomarkers	Neurocognitive association
Auvinen et al. (2022)	180 newborns (80 PAE/100 controls); mothers from substance abuse clinic	Placenta, umbilical cord WBCs, buccal epithelial cells	Epigenetic (DNAm, histone marks)	DNAm in *DPPA4, FOXP2, TACR3*; H3K4me2/3, H3K9ac	Alterations in neurodevelopmental pathways (axon guidance, synapse organization)
Lussier et al. (2018)	48 children (24 FASD/24 controls), 3.5–18 years	Buccal epithelial cells	Epigenetic (DNAm)	DNAm signature (648 CpGs; e.g., *SLC6A3, HLA-DPB1, H19*)	Associated with FASD diagnosis and cognitive/adaptive deficits, independent of physical features
Van der Laan et al. (2025)	93 individuals with suspected/confirmed FASD (0.4–37 years)	Peripheral blood	Epigenetic (DNAm episignature)	204 CpG probes (52% hypermethylated)	DNAm profiles correlate with cognitive and behavioral impairment
Krzyzewska et al. (2023)	63 individuals (12 severe FASD/51 controls)	Whole blood	Epigenetic (eQTM)	6 eQTMs (*SEC61G, REEP3, ZNF577, HNRNPF, MSC, SDHAF1*)	Genes linked to intellectual disability, ADHD, and neurodevelopmental disorders
Bakhireva et al. (2025)	100 mother–infant dyads (32 PAE/68 controls) (ENRICH-2 cohort)	Placenta, cord blood	Endocrine (HPA-axis)	*NR3C1, HSD11B2, pCRH;* cortisol/cortisone	Placental and cord blood markers associated with Bayley-III cognitive, language, and motor scores and infant temperament at 6–9 months
Deyssenroth et al. (2024)	62 mother–infant dyads (32 heavy PAE/30 controls) (cape town cohort)	Placenta	Transcriptomic (RNA-seq, WGCNA)	Gene networks (erythropoiesis, angiogenesis)	Placental gene networks associated with PAE and developmental pathways relevant to neurodevelopment
Williams et al. (2024)	68 mother–infant dyads (35 heavy drinkers/33 controls) (cape town cohort)	Placenta	Inflammatory gene expression	*NOS2, PTGER3*; increased hofbauer cells	Pro-inflammatory placental environment linked to later neurodevelopmental delay
Holbrook et al. (2019)	26 mother–infant dyads (13 PAE/13 controls) (ENRICH cohort)	Placenta	Angiogenic proteins	VEGFR2, ANXA4 (trends: VEGFR1, CCM3)	Impaired placental vascularization associated with restricted fetal brain growth
Darbinian et al. (2024)	80 pregnant women (40 PAE/40 controls)	Maternal plasma (FBDEs)	miRNAs (exosomal)	miR-9 and miR-132	Reductions in FB-E miR-9 levels correlated strongly with reductions in fetal eye diameter
Mahnke et al. (2021)	68 infants (37 heavy PAE/31 controls) (cape town cohort)	Plasma (2 weeks, 6.5 months)	Circulating miRNAs	miR-21, miR-92a, miR-126, miR-146a, miR-328	miRNA profiles associated with growth restriction and lower Bayley-III scores
Almeida et al. (2025)	9 newborns (4 PAE/5 controls)	Umbilical cord serum	Proteomic	APOA4, immune and vascular proteins	BBB dysfunction and neuroinflammatory vulnerability
Andreu-Fernández et al. (2019)	150 children (86 FASD/33 PAE/31 controls)	Serum	Growth factors	IGF-I, IGF-II	Lower levels associated with reduced IQ and growth retardation
Bodnar et al. (2020)	59 infants (32 PAE/27 controls)	Plasma	Immune network analysis	Cytokine networks	Immune network dysregulation associated with neurodevelopmental delay in PAE-exposed infants
Maxwell et al. (2024)	18 newborns (pilot study; 8 PAE/10 controls) (ENRICH-2 cohort)	Umbilical cord PBMCs	Innate immune response	TLR-mediated cytokine responses (TLR2, TLR3, TLR4, TLR9 stimulation)	Enhanced cytokine responses suggest immune priming associated with altered neurodevelopmental vulnerability

Abbreviations: ADHD, attention-deficit/hyperactivity disorder; APOA4, apolipoprotein A-IV; BBB, blood–brain barrier; BEC, buccal epithelial cells; CpG, cytosine–phosphate–guanine dinucleotide; DNAm, DNA, methylation; eQTM, expression quantitative trait methylation; FAS, fetal alcohol syndrome; FASD, fetal alcohol spectrum disorder; FBDEs, fetal brain–derived exosomes; HPA axis, hypothalamic–pituitary–adrenal axis; HP, haptoglobin; IGF-I/II, insulin-like growth factor I/II; IL, interleukin; miRNA, microRNA; PAE, prenatal alcohol exposure; PBMCs, peripheral blood mononuclear cells; PlGF, placental growth factor; RA, retinoic acid; RNA-seq, RNA, sequencing; SNP, single nucleotide polymorphism; TLR, toll-like receptor; TNF-α, tumor necrosis factor alpha; VEGF-A, vascular endothelial growth factor A; VEGFR1, vascular endothelial growth factor receptor 1; WBCs, white blood cells; WGCNA, weighted gene co-expression network analysis.

Across independent human cohorts, these studies describe a multilayered molecular landscape encompassing stable epigenetic modifications, coordinated placental transcriptomic and proteomic alterations, dysregulated endocrine and immune signaling, and circulating RNA signatures. The findings presented below are organized by biological regulatory level and tissue of origin, focusing on biomarkers measured in placenta, umbilical cord blood, and peripheral biological fluids in relation to neurodevelopmental outcomes following PAE.

### 2.1 Epigenetic biomarkers of PAE

At the level of long-term molecular programming, multiple human studies have identified DNA methylation (DNAm)-based biomarkers associated with PAE across placental tissue and accessible peripheral samples, with recurrent involvement of genes regulating pluripotency, neurodevelopment, growth, and cellular stress responses.

Placental epigenome-wide association analyses revealed extensive DNAm alterations associated with PAE. Auvinen et al. identified 689 differentially methylated CpG sites and 112 differentially methylated regions (DMRs) in placental tissue from alcohol-exposed newborns, predominantly characterized by hypomethylation (Auvinen et al., 2022). DMRs were enriched in genes involved in early developmental regulation and neurodevelopment, including *DPPA4*, a chromatin modifier regulating pluripotency and lineage commitment; *FOXP2*, a transcription factor critical for speech, language, and cortical development; and *TACR3*, involved in neuroendocrine and hypothalamic signaling. Additional differentially methylated loci were detected at imprinting control regions regulating *H19* and *IGF2*, genes essential for fetal growth and brain development. DNAm levels at these imprinting regions were significantly associated with reduced birth weight and head circumference, and surrogate markers of impaired fetal brain growth. Timing analyses demonstrated that periconceptional alcohol exposure was associated with the largest magnitude of DNAm change. In addition, the authors explored whether selected placental DNAm alterations could also be detected in umbilical cord white blood cells and buccal epithelial cells. While the majority of alcohol-associated DNAm changes were tissue-specific, differential methylation at *DPPA4* showed consistent directionality across placenta, cord blood, and buccal epithelial cells, suggesting that a subset of placental epigenetic alterations may be detectable in clinically accessible peripheral tissues.

Peripheral DNAm biomarkers were further identified in buccal epithelial cells. Lussier et al. described a DNAm “neurosignature” consisting of 648 CpG sites that robustly discriminated individuals with FASD from unexposed controls across independent cohorts (Lussier et al., 2018). Differentially methylated CpGs mapped to genes involved in dopaminergic neurotransmission (*SLC6A3*), immune regulation (*HLA-DPB1, HLA-DQA1*), growth and imprinting (*H19*), neuronal differentiation, axon guidance, and synaptic signaling. This DNAm neurosignature demonstrated high sensitivity and specificity and was shown to be distinct from DNAm patterns observed in autism spectrum disorder, supporting its specificity for FASD.

Blood-based DNAm episignatures further extended these findings. Van der Laan et al. identified a DNA methylation episignature comprising 204 CpG sites that accurately classified individuals with clinically confirmed FASD across independent cohorts spanning infancy, childhood, adolescence, and adulthood (van der Laan et al., 2025). Differentially methylated loci included *REEP3*, involved in endoplasmic reticulum organization; *HNRNPF*, a regulator of RNA splicing and processing; *SDHAF1*, linked to mitochondrial function; *MSC*, a transcription factor involved in craniofacial and neural crest development; and *TNFRSF19*, associated with neuronal apoptosis. Classification performance remained stable when the episignature was validated in independent cohorts and across different DNA methylation array platforms, supporting its reproducibility and technical robustness.

Functional relevance of DNAm alterations was supported by integrative analyses. Krzyzewska et al. performed expression quantitative trait methylation (eQTM) analysis in whole blood and identified cis-regulatory relationships between DNAm and gene expression for *HNRNPF*, *REEP3*, *SEC61G*, *SDHAF1*, and *MSC* (Krzyzewska et al., 2023). Variation in DNAm at these loci was associated with corresponding changes in gene expression and showed associations with clinical neurodevelopmental phenotypes, including intellectual disability and attention-deficit/hyperactivity disorder.

### 2.2 Placental transcriptomic and cellular biomarkers

Beyond epigenetic regulation, transcriptomic analyses of human placenta at birth have identified alcohol-associated gene expression signatures and changes in placental cellular composition. These alterations implicate pathways related to oxygen transport, vascular development, immune signaling, and extracellular matrix organization.

Bulk RNA sequencing analyses demonstrated coordinated disruption of placental gene expression networks following PAE. Deyssenroth et al. identified 40 differentially expressed genes and multiple gene co-expression modules associated with PAE (Deyssenroth et al., 2024). Modules most strongly associated with PAE were enriched for genes involved in erythrocyte differentiation and heme biosynthesis (*TRIM58*, *ALAS2*, *HBA1*, *HBA2*), angiogenesis and hypoxia response (*ETS1*, *KDR*, *EGLN1*), extracellular matrix remodeling, and neutrophil-mediated immunity. These transcriptomic alterations were most pronounced following periconceptional exposure and became evident only after adjustment for placental cell-type composition, underscoring the importance of cell-type–aware analyses.

Cell-type–resolved transcriptomic analyses further demonstrated immune-related placental alterations. Williams et al. reported increased proportions of Hofbauer cells, the resident fetal macrophages of the placenta, in alcohol-exposed pregnancies (Williams et al., 2024). After accounting for cellular composition, differential expression of 35 inflammation-related genes was observed, including *NOS2*, *PTGER3*, *IL1RN*, *IL17D*, *CCR2*, *XCR1*, *CRH*, and *LEP*. These genes are involved in nitric oxide signaling, prostaglandin pathways, cytokine and chemokine regulation, and neuroendocrine signaling.

### 2.3 Endocrine and growth factor biomarkers

Endocrine and growth factor-related biomarkers measured in placental tissue, cord blood, and peripheral circulation have been associated with PAE and early neurodevelopmental outcomes.

In participants from the Ethanol, Neurodevelopment, Infant and Child Health (ENRICH-2) prospective cohort with documented PAE, placental biomarkers related to hypothalamic–pituitary–adrenal (HPA) axis regulation predicted infant neurodevelopment. The study integrated placental gene expression, protein levels, and cord blood endocrine measures to examine associations with developmental outcomes (Bakhireva et al., 2025). Expression of *NR3C1*, encoding the glucocorticoid receptor, and *HSD11B2*, encoding 11β-hydroxysteroid dehydrogenase type 2, differed according to PAE status. Protein levels of 11β-HSD2 and the 11β-HSD2/11β-HSD1 ratio, indicative of placental cortisol inactivation capacity, were also quantified. These glucocorticoid-related markers showed significant associations with multiple domains of neurodevelopment assessed using the Bayley Scales of Infant and Toddler Development at 6–9 months of age, including cognitive, language, and motor composite scores. Higher *HSD11B2* expression and higher 11β-HSD2/11β-HSD1 ratios were associated with more favorable developmental performance. Placental corticotropin-releasing hormone (pCRH) concentrations were additionally associated with infant temperament dimensions, including orienting/regulation and negative affect, assessed using standardized behavioral questionnaires (Bakhireva et al., 2025).

Cord blood endocrine measures provided complementary information. Cortisol and cortisone concentrations measured at birth were associated with placental glucocorticoid-related biomarkers and contributed to multivariable models predicting infant neurodevelopmental outcomes. Associations were observed for both cognitive and motor domains.

Alterations in growth factor signaling were identified in peripheral circulation beyond the neonatal period. Andreu-Fernández et al. quantified serum insulin-like growth factors IGF-I and IGF-II in children with documented PAE and FASD across childhood (Andreu-Fernández et al., 2019). Both IGF-I and IGF-II concentrations were significantly reduced in exposed children compared with controls. IGF-II levels showed significant associations with multiple neurocognitive domains, including full-scale intelligence quotient, working memory, processing speed, and verbal comprehension. In contrast, IGF-I concentrations were more strongly associated with somatic growth parameters, including height, weight, and head circumference. These associations remained significant after adjustment for age and sex.

### 2.4 Immune and inflammatory biomarkers

Immune-related biomarkers measured in umbilical cord blood, placental tissue, and peripheral circulation revealed altered innate immune responsiveness following PAE.

A recent pilot study by Maxwell et al. (2024) demonstrated that moderate PAE is associated with increased Toll-like receptor (TLR) activity in umbilical cord blood. Specifically, umbilical cord blood-derived peripheral blood mononuclear cells (PBMCs) from PAE-exposed newborns exhibited enhanced cytokine responses following TLR stimulation, suggesting an immune priming effect that may increase neurodevelopmental vulnerability later in life (Maxwell et al., 2024).

In early childhood, plasma cytokine network analyses revealed coordinated immune alterations. Bodnar et al. identified immune interaction networks involving IL-6, TNF-α, C-reactive protein, monocyte chemoattractant protein-4, macrophage-derived chemokine, macrophage inflammatory protein-1β, and placental growth factor that were differentially organized in alcohol-exposed children with neurodevelopmental delay compared with those showing relative resilience (Bodnar et al., 2020). These immune network profiles were associated with cognitive development scores.

Placental immune alterations provide a tissue-level correlate to these systemic immune findings. Transcriptomic analyses of alcohol-exposed placentas revealed upregulation of inflammation-related genes, including *NOS2*, *PTGER3*, *IL1RN*, *CCR2*, and *IL17D*, in association with altered placental immune cell composition (Williams et al., 2024). These placental immune signatures parallel the structured cytokine network alterations observed in cord blood and early childhood plasma, supporting the presence of coordinated immune dysregulation across the fetoplacental and peripheral compartments.

### 2.5 Circulating RNA and extracellular vesicle biomarkers

Circulating RNA biomarkers measured at different developmental stages provide complementary information on molecular alterations associated with PAE. Mahnke et al. analyzed circulating microRNAs in infant plasma collected at 2 weeks and 6.5 months of age and identified coordinated changes in microRNA expression associated with postnatal growth and neurodevelopmental outcomes (Mahnke et al., 2021). Differentially expressed microRNAs included miR-21-5p, miR-92a-3p, miR-126-3p, miR-146a-5p, miR-328-3p, and miR-29a-3p, which are involved in angiogenesis, immune regulation, cell proliferation, and neuronal development. Importantly, microRNA expression patterns measured at 2 weeks of age were significantly associated with cognitive performance assessed at 12 months, as well as with head circumference and weight trajectories during infancy, indicating persistence of prenatal molecular programming into the postnatal period.

Extracellular vesicle–associated RNA biomarkers provided additional specificity for fetal brain–derived molecular signals during gestation. Darbinian et al. isolated fetal brain–derived exosomes from maternal blood using neuron-specific markers and quantified their microRNA cargo during pregnancy (Darbinian et al., 2024). This approach enabled selective assessment of extracellular vesicles originating from the developing fetal brain. Alcohol-exposed pregnancies exhibited significantly reduced levels of miR-9 and miR-132 within these exosomes. Both microRNAs are key regulators of neuronal differentiation, synaptic plasticity, and activity-dependent gene expression. Altered exosomal microRNA levels were significantly associated with reduced fetal eye diameter, a surrogate marker of early brain development, and with changes in downstream protein targets involved in neurogenesis, axonal development, and synapse formation.

### 2.6 Proteomic and vascular-related biomarkers

Proteomic analyses of placental tissue and umbilical cord blood have identified alcohol-associated alterations in proteins involved in angiogenesis, vascular integrity, inflammation, and barrier-related processes. In placental tissue, Holbrook et al. quantified the expression of vascular and inflammation-related proteins and reported significantly reduced levels of angiogenesis-associated markers in alcohol-exposed pregnancies, including vascular endothelial growth factor receptor 2 (VEGFR2) and annexin A4 (ANXA4), with additional trends toward reduced expression of VEGFR1 (FLT1) and cerebral cavernous malformation protein 3 (CCM3) (Holbrook et al., 2019). These proteins play key roles in endothelial proliferation, vascular stabilization, and microvascular organization, suggesting that PAE disrupts placental vascular signaling and inflammatory balance.

Complementary proteomic profiling of umbilical cord blood serum revealed a distinct circulating protein signature in alcohol-exposed neonates. Almeida et al. identified altered levels of apolipoproteins, immune-related proteins, and angiogenic regulators using an unbiased proteomic approach (Almeida et al., 2025). Among these, apolipoprotein A-IV (APOA4) was significantly reduced in exposed neonates. Functional assays demonstrated that exposure of human brain microvascular endothelial cells to serum from alcohol-exposed neonates resulted in increased endothelial permeability and impaired cell migration, accompanied by reduced expression of tight junction proteins and angiogenic factors. Together, these findings suggest that circulating proteomic alterations associated with PAE may reflect early disruptions in vascular and barrier-related processes relevant to brain development.

## 3 Discussion

This Mini Review synthesizes recent human evidence identifying molecular biomarkers associated with neurodevelopmental alterations following PAE. Across independent cohorts and heterogeneous study designs, convergent findings emerge at multiple biological levels, including epigenetic regulation, placental transcriptomic and proteomic profiles, endocrine and immune signaling, and circulating RNA and protein biomarkers. Collectively, these data support the concept that PAE leaves a persistent and biologically coherent molecular footprint. This molecular footprint can be detected in the placenta and in clinically accessible biological fluids and is associated with neurodevelopmental outcomes across early life.

Importantly, the identification of such biomarkers is not merely descriptive but directly aligned with the need for early detection of neurodevelopmental vulnerability in children exposed to alcohol *in utero*. Given that PAE is frequently underrecognized and that many children lack overt physical features of FASD, molecular biomarkers may provide an objective tool to identify at-risk infants before the full clinical phenotype becomes apparent. This is particularly relevant in contexts where alcohol consumption during pregnancy is underestimated or perceived as low risk.

This review summarizes the current evidence in biological systems with the aim of contributing to the development of biomarker-based strategies capable of supporting early risk stratification and timely intervention. Although further validation studies and clinical trials are required before routine implementation, the data reviewed here provide a structured framework for advancing translational research focused on mitigating long-term neurodevelopmental consequences of PAE.

### 3.1 Convergence of biomarker signals across biological systems

A central finding across the reviewed studies is the convergence of biomarker alterations on biological pathways regulating early brain development, growth control, and stress responsivity. Across epigenetic, transcriptomic, and proteomic analyses, recurrent signals implicate genes involved in developmental programming, imprinting control, neuroendocrine regulation, and cellular differentiation (Lussier et al., 2018; Holbrook et al., 2019; Auvinen et al., 2022; Krzyzewska et al., 2023; Deyssenroth et al., 2024; Williams et al., 2024; van der Laan et al., 2025), supporting coordinated disruption of regulatory systems following PAE.

Epigenetic investigations consistently identify differential DNA methylation in genes governing pluripotency, neurodevelopmental transcriptional control, and growth signaling. The partial cross-tissue detectability of selected methylation marks suggests that developmental reprogramming events occurring in the placenta may, in some cases, be traceable in peripheral tissues (Lussier et al., 2018; Auvinen et al., 2022; Krzyzewska et al., 2023; van der Laan et al., 2025). Notably, many of these alterations map to pathways with established roles in neuronal differentiation, synaptic development, and hypothalamic-pituitary-adrenal axis regulation, reinforcing their biological relevance.

Placental transcriptomic and proteomic findings further demonstrate disruption of networks related to angiogenesis, oxygen transport, and immune regulation (Holbrook et al., 2019; Deyssenroth et al., 2024; Williams et al., 2024). Evidence of immune cell remodeling within the placenta indicates that inflammatory processes are embedded within the fetoplacental interface following PAE. Collectively, these data position the placenta not only as a record of exposure but as an active biological interface linking PAE to downstream neurodevelopmental vulnerability.

### 3.2 Endocrine and immune biomarkers as functional correlates of neurodevelopment

Endocrine and immune biomarkers are among the most clinically informative findings, as they reflect functional systems directly involved in stress regulation and brain maturation. Alterations in placental and cord blood markers related to glucocorticoid signaling point to dysregulation of the hypothalamic–pituitary–adrenal axis following PAE (Bakhireva et al., 2025). Rather than isolated hormonal changes, these alterations suggest recalibration of fetal stress responsivity during sensitive developmental windows, with downstream implications for early cognitive and behavioral outcomes.

From a developmental perspective, glucocorticoids are key regulators of neuronal maturation and stress circuitry. Disruption of placental glucocorticoid regulation may constitute a shared pathway linking PAE to altered stress responsivity. These findings support the concept of the placenta as an active mediator of prenatal stress programming rather than a passive record of exposure.

Parallel evidence from immune studies further supports the concept of functional system-level alteration. Immune biomarkers measured at birth and during early childhood reveal altered innate immune responsiveness rather than overt baseline inflammation (Bodnar et al., 2020; Maxwell et al., 2024). This pattern is consistent with immune reprogramming or priming, in which cytokine responses to stimulation are modified without necessarily elevating resting inflammatory levels. The identification of coordinated cytokine interaction networks associated with neurodevelopmental delay indicates that immune dysregulation following PAE is structured and network-based, rather than attributable to single mediators.

Importantly, the overlap between placental immune gene expression changes and peripheral immune signatures suggests that endocrine and immune pathways may represent interconnected arms of a broader stress-regulatory axis. Given the well-established bidirectional communication between glucocorticoid signaling and immune activation, these findings raise the possibility that PAE induces a persistent shift in neuroimmune set points during development. Such integrated dysregulation may represent a mechanistic bridge linking early-life alcohol exposure to later vulnerability to cognitive and emotional disorders. Therefore, identifying early inflammatory biomarkers may help identify individuals at increased risk and support earlier preventive strategies.

Notably, sex-dependent differences in immune responsiveness have been reported in neonates with PAE (Maxwell et al., 2024). Such findings suggest that biological sex may modulate early immune programming and stress-axis regulation following PAE. Incorporating sex as a biological variable in future biomarker studies may therefore improve risk stratification and enhance understanding of differential vulnerability trajectories across development.

### 3.3 Circulating RNA and proteomic biomarkers: toward minimally invasive detection

RNA-based and proteomic biomarkers represent a particularly promising avenue for clinical translation, as they can be obtained through minimally invasive sampling and may capture dynamic aspects of neurodevelopmental programming. Circulating microRNAs measured in infant plasma shortly after birth have been shown to predict later cognitive performance and growth trajectories (Mahnke et al., 2021), suggesting that molecular alterations established during prenatal life remain biologically traceable in the early postnatal period. Importantly, microRNAs regulate gene expression networks involved in angiogenesis, immune signaling, and neuronal differentiation, positioning them as upstream modulators rather than passive markers of injury.

The identification of fetal brain-derived exosomal microRNAs in maternal blood during pregnancy further strengthens the translational potential of RNA-based biomarkers (Darbinian et al., 2024). Because these exosomes originate from fetal neural tissue, their molecular cargo may provide a more direct window into *in utero* neurodevelopmental processes than peripheral markers alone. This approach moves beyond exposure detection and begins to approximate real-time monitoring of fetal neurobiological status, an advance with clear implications for early risk stratification.

Proteomic profiling of umbilical cord blood expands this minimally invasive framework by identifying alterations in circulating proteins linked to vascular integrity, angiogenesis, and endothelial function (Almeida et al., 2025). Notably, the functional assays demonstrating endothelial barrier disruption following exposure to serum from alcohol-exposed neonates suggest that these proteomic signatures may reflect biologically active systemic alterations rather than incidental correlates. Vascular development and blood–brain barrier maturation are critical for shaping the neural microenvironment. Therefore, these findings provide a mechanistic bridge between peripheral protein alterations and central neurodevelopmental vulnerability.

Taken together, circulating RNA and proteomic biomarkers may capture complementary dimensions of early-life programming: regulatory (microRNA-mediated gene control), inflammatory and vascular (protein-based signaling), and tissue-specific (exosome-derived neural cargo). Their accessibility across prenatal and early postnatal stages makes them particularly attractive candidates for longitudinal monitoring and for integration into multimodal biomarker panels aimed at early identification of children at risk for neurodevelopmental impairment following PAE.

### 3.4 Methodological challenges and research gaps

Despite substantial progress, important methodological challenges limit the immediate clinical translation of currently identified biomarkers. Sample sizes across studies are frequently modest, and cohort heterogeneity in exposure characterization, timing of alcohol consumption, and diagnostic classification introduces variability that complicates direct comparison across investigations. As summarized in Table 1, studies include diverse cohort designs ranging from prospective cohorts such as ENRICH and the Cape Town birth cohort to smaller clinical or case–control studies, with alcohol exposure patterns varying from moderate prenatal exposure to heavy exposure or clinically diagnosed FASD. Differences in biospecimen type (placenta, cord blood, buccal cells, plasma), analytical platforms (array-based DNAm, RNA sequencing, targeted protein assays), and neurodevelopmental outcome measures further reduce cross-study harmonization. In addition, the generalizability of these findings may be influenced by the geographic and demographic characteristics of the cohorts studied. Several investigations derive from specific regional cohorts, including populations in North America, South Africa, and Eastern Europe, and often involve socially vulnerable groups. While some studies include participants from diverse backgrounds (Maxwell et al., 2024), large multi-ethnic cohorts remain limited. In addition, few studies are sufficiently powered to systematically evaluate sex-specific effects, despite emerging evidence that biological sex may influence immune responsiveness and stress-axis regulation following PAE.

A critical limitation is the predominance of single time-point assessments. Most studies evaluate biomarkers at birth or during early infancy without repeated sampling, limiting the ability to characterize dynamic trajectories or to distinguish transient exposure signatures from persistent molecular programming. Given that neurodevelopment unfolds across multiple sensitive windows, longitudinal biomarker profiling is essential to determine stability, evolution, and predictive value over time.

Another gap concerns multimodal integration. Although epigenetic, endocrine, immune, RNA-based, and proteomic alterations have each been independently associated with neurodevelopmental outcomes, relatively few studies have examined these domains concurrently within the same individuals. As a result, it remains unclear whether these biomarkers reflect shared upstream mechanisms, parallel but independent pathways, or partially overlapping biological processes. System-level integration approaches, including network-based and multi-omics analyses, may be required to define composite biomarker signatures with improved specificity and predictive performance.

Importantly, while many biomarkers demonstrate statistically significant associations with neurodevelopmental outcomes, causality cannot be inferred in human observational cohorts. Residual confounding, co-exposures (e.g., tobacco, psychosocial stress), and postnatal environmental influences may contribute to observed molecular patterns. Carefully designed longitudinal studies with standardized neurodevelopmental assessments, detailed exposure quantification, and replication across independent populations will be essential to establish temporal directionality and to determine whether identified biomarkers possess true prognostic value.

### 3.5 Implications for future biomarker-driven strategies

Future research should prioritize integrative and longitudinal designs combining epigenetic, immune, endocrine, vascular, and RNA-based biomarkers across developmental stages. Multimodal panels are likely to provide a more robust characterization of neurodevelopmental risk and resilience following PAE than single molecular indicators. Standardization of exposure assessment, biospecimen collection, analytical pipelines, and neurodevelopmental outcome measures will be essential to enable cross-cohort validation and improve reproducibility.

From a translational perspective, minimally invasive biomarkers detectable in placenta, cord blood, and peripheral fluids may help address the frequent underrecognition of PAE-related neurodevelopmental impairment, which often emerges progressively and may occur without overt physical features (Mattson et al., 2011; Cook et al., 2016; Roozen et al., 2016; May et al., 2018). Evidence that even low-to-moderate exposure is associated with measurable neurobehavioral alterations further supports the importance of identifying vulnerability at stages when intervention may still influence developmental trajectories (Lees et al., 2020; Maxwell et al., 2025).

Although clinical implementation remains premature, convergent findings across independent cohorts suggest that molecular biomarkers could support earlier risk stratification. Progress toward validated, longitudinally predictive biomarker frameworks will be critical for translating developmental programming research into clinically actionable strategies in the context of PAE.
